# Delirium in Cardiac Intensive Care Unit

**DOI:** 10.7759/cureus.10096

**Published:** 2020-08-28

**Authors:** Sukrut Pagad, Manoj R Somagutta, Vanessa May, Ashley A Arnold, Saruja Nanthakumaran, Saijanakan Sridharan, Bilal Haider Malik

**Affiliations:** 1 Department of Research, California Institute of Behavioral Neurosciences & Psychology, Fairfield, USA; 2 Department of Research, California Institute of Behavioral Neurosciences & Psychology, Fairfield, CAN; 3 Internal Medicine, California Institute of Behavioral Neurosciences & Psychology, Fairfield, USA

**Keywords:** delirium, cardiac, icu

## Abstract

Delirium is a multifactorial syndrome and is described as an acute brain dysfunction seen commonly in post-cardiac surgery patients. The prevalence of post-operative Delirium (POD) ranges from 11.4% to 55%, depending on the diagnostic tool and type of study. Confusion Assessment Method for the Intensive Care Unit (CAM-ICU) and the Intensive Care Delirium Screening Checklist (ICDSC) are the two most used and recommended tools by the Society of Intensive Care Medicine. Annual delirium-related healthcare costs in the United States (US) range from 6.6 to 20.4 billion USD in ICU patients. However, delirium in cardiac ICU (CICU) is underdiagnosed and warrants vigorous workup. The risk factors for delirium in CICU can be classified as modifiable, non-modifiable, and cardiac surgical causes. After cardiac procedures, delirium is associated with increased mortality, increased length of hospital stay, loss of functional independence, increased hospital costs, and an independent predictor of death 10 years postoperatively. Non-pharmacological measures such as avoiding delirium-risk medications, early physical rehabilitation, occupational therapy, and sleep improvement strategies have shown significant benefits in decreasing delirium. Pharmacological options are limited for use in CICU, and a need for future studies in this topic is in demand.

## Introduction and background

According to the Diagnostic and Statistical Manual of Mental Disorders (DSM 5), delirium is characterized by a disturbance in attention (i.e., reduced ability to direct, focus, sustain, and shift attention), awareness (reduced orientation to the environment) or cognition (e.g., disorientation, language, visuospatial ability, or perception) [1]. The prevalence of post-operative delirium (POD) varies from 11.4% to 55%, depending on the diagnostic tool and type of study [2,3]. According to American Psychiatric Association, the diagnosis needs to be supplemented by observational assessment tools like Confusion Assessment Method (CAM), Confusion Assessment Method - Intensive care unit (CAM-ICU), and Intensive Care Delirium Screening Checklist (ICDSC). These assessment tools will help improve the identification of delirium compared to clinical judgment alone [4].

Despite the many complications, the exact pathophysiology behind POD is unknown though some speculate it could be multifactorial; the effects of anesthesia used during surgery, disruption of the blood-brain barrier due to inflammatory chemokines, decreased cerebral oxygenation, or medications consumed before surgery could contribute [5,6]. Delirium is a well-recognized syndrome seen in patients admitted to the medical ward with an illness, ICU, and cardiac surgical wards [7,8]. It is an acute transient state that can lead to serious adverse effects if left untreated. Postoperatively, in cardiac patients, delirium is described as acute brain dysfunction. It is associated with increased mortality, increased length of hospital stay, loss of functional independence, increased hospital costs, and, according to Gottesam et al., an independent predictor of death 10 years postoperatively [2,9,10].

Early identification of POD is of utmost importance for the patient’s safety. Therefore, this review article aims to provide an overview of the risk factors and prevalence of POD in cardiac surgical patients and suggest appropriate management options. Summarizing current scientific evidence and reports will help clinicians hypothesize possible strategies to prevent post-operative delirium.

## Review

Diagnosis and types

According to the DSM-5, delirium is defined as a change in mental status, inattention, disorientation, disorganized thinking, disturbed sleep-wake cycle, and altered level of consciousness [1]. The onset of postoperative delirium is frequently acute, mainly one to three days post-surgery. The notable subtypes of delirium are hyperactive, hypoactive, and mixed forms [11] with varying symptom presentations, as demonstrated in Table 1. The hypoactive being the most common form of delirium as high as 92% in one study, is also associated with increased mortality as it is challenging to diagnose in earlier stages [12]. Post-cardiac surgery delirium is identified as a significant postoperative neurologic complication [13]. The incidence of delirium ranges widely (from 3% to 52%) in post-cardiac surgery patients depending on the methodology, screening tools, and the type of study [14,15]. POD is associated with cognitive decline, prolonged hospital stay, increased hospital costs, institutionalization, physical and emotional stress. Importantly, these patients have a higher risk of mortality within the first years after cardiac surgery [11,16].

**Table 1:** Summary of the types of delirium

Delirium type	Symptoms	Resembles
Hyperactive Delirium	1. Hypervigilance 2. Increased arousal 3. Increased psychomotor activity 4. Agitation 5. Restlessness	1. Manic 2. Psychotic decompensation
Hypoactive Delirium	1. Withdrawn 2. Lethargic 3. Decreased motor activity 4. Apathy 5. Decreased responsiveness	Depression
Mixed motor type	Combination of the above	Rapid cycling

Pathogenesis

The exact pathophysiology behind POD is poorly understood, although many recent studies have attempted to give independent mechanisms that may play a role in the act. However, a spectrum of factors may be responsible for delirium onset [17]. Possible pathways for POD include increased systemic inflammation, changes in neurotransmitters, especially acetylcholine, intraoperative changes, and genetic factors [18-20]. Recent studies exhibit the vital role of microglial activation mediates the pro-inflammatory response, subsequently leading to intense neurological inflammation resulting in POD in cardiac surgery patients [6,17,21].

Risk factors

**Figure 1:**
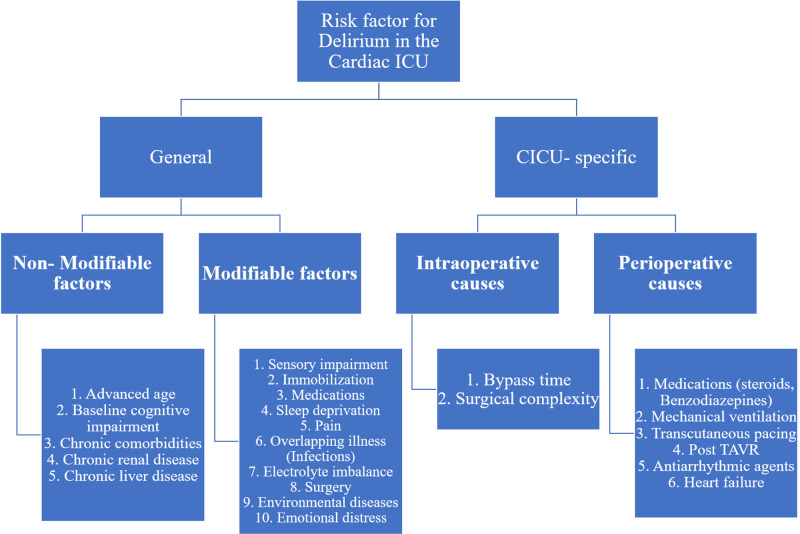
Risk factors for delirium in the cardiac ICU ICU: Intensive Care Unit, CICU: Cardiac Intensive Care Unit, TAVR: Transcatheter aortic valve replacement

Factors involving postoperative delirium (POD), especially related to cardiac surgery, are multifactorial. Intraoperative changes such as cardiac dysfunction, susceptibility to cardiopulmonary bypass, microemboli, rise in the internal body temperature, intravenous pH paired with changes in the cerebral oxygenation, anesthetics, and cerebral inflammation may precipitate delirium in high-risk individuals [5]. Listed in Figure 1 are the various risk factors associated with delirium seen in patients admitted to the cardiac ICU. 

Some authors correlate the systemic inflammatory response, primarily triggered by cardiopulmonary bypass or generation of air microembolism during open-heart surgery, which has an incremental effect in neuropsychological stress response leads to POD [13,22]. Some other authors associate it with advanced atherosclerosis and endothelial dysfunction as a primary source of increased cerebral microembolization risk [13,23].

Intraoperative oxidative injury contributes to oxidative stress, endothelial damage, and acute kidney injury (AKI) [19]. Lopez et al. conducted a prospective study to identify the link between intraoperative hyperoxic cerebral reperfusion and POD. The results infer that intraoperative hyperoxic cerebral reperfusion was independently associated with a 65% increase in the odds of delirium (odds ratio [OR], 1.65 (1.12 to 2.44); P=0.01) [5]. 

Few studies have been done to evaluate the interplay of cerebral flow oxygen and delirium. Imaging techniques such as single-electron emission computed tomography and near-infrared spectroscopy (NIRS), a non-invasive device, are used to measure cerebral brain oxygenation (cerebral oximetry) [6]. This measure also indicates cerebral blood flow; hence it is hypothesized that it may be a possible monitor of delirium.

In a prospective observational study by Mailhot et al., post-cardiac delirium patients are monitored with NIRS three consecutive days after the onset. The authors noticed that the cerebral oximetry levels decreased with the start of delirium and raised back once delirium resorbed. Thus, making cerebral oximetry associated with delirium diagnosis and severity, but not peripheral oximetry [6]. 

Screening

Despite the frequent occurrence of delirium in the CICU, it is often underdiagnosed there, which indicates the necessity of standard evaluation in all critically ill patients [24]. In the CICU, screening for delirium requires a more collaborative approach from the cardiologists, nurses, and ICU physicians and other healthcare personnel involved in the patient's care [25].

Generally, the ICU patients may be sedated, intubated, and comparatively sick than non-ICU patients demanding a vigorous workup for delirium. Various screening tools are used in delirium screening in ICU. The commonly used tools are Intensive Care Delirium Screening Checklist (ICDSC), the Confusion Assessment Method for the Intensive Care Unit (CAM-ICU), the Nursing Delirium Screening Scale (Nu-DESC), the Delirium Detection Score (DDS), Diagnostic and Statistical Manual IV-TR (DSM IV-TR) and the Cognitive Test for Delirium (CTD) [26]. But CAM-ICU and ICDSC are the two most used and recommended tools by the Society of Intensive Care Medicine [25].

In a meta-analysis (including nine studies and 969 patients), CAM-ICU and ICDSC were analyzed for their effectiveness in screening delirium. The authors observed that the sensitivity of the CAM-ICU was 80.0% (95% CI: 77.1 to 82.6%) and the specificity was 95.9% (95% CI: 94.8 to 96.8%). The sensitivity of the ICDSC was 74% (95% CI: 65.3 to 81.5%), and the specificity was 81.9% (95% CI: 76.7 to 86.4%) [27]. In another prospective cohort study, CAM-ICU and ICDSC were evaluated versus the DSM-IV-TR in delirium diagnosis concerning their validity and psychometric properties. They noticed CAM-ICU showed only modest concurrent validity (Cohen's κ = 0.44) and sensitivity (50%) but high specificity (95%). The ICDSC attained a sensitivity of 63% and specificity of 95%. Within the CAM-ICU and the ICDSC, ICDSC produced greater sensitivity and specificity (78 and 83%) with a moderate validity (Cohen's κ = 0.56) compared to CAM-ICU [28]. Thus, a negative test by the CAM-ICU or ICDSC should not negate delirium, and additional workup is required, especially in high-risk patients.

Non-pharmacological measures

Annual delirium-related healthcare costs in the United States (US) are estimated to range from 6.6 to 20.4 billion USD in ICU patients and 38 to 152 billion USD per year in non-ICU patients. As a result, the focus of management is effective prevention, as therapeutic options are limited. Several management guidelines and standardized programs were developed to prevent, early recognition, and treatment of delirium across ICU and non-ICU settings [29,30]. Cost-effective interventions such as setting up clocks and calendars, reminding patients on the current time, place, and date, sleep support by avoiding late-night medication administration, less stressful and quiet environment, and early mobilization are proven to be useful in the non-ICU situations. Cybertherapy or hypnosis, massage, music therapy, cold therapy, relaxation techniques are all recommended for pain management in critically ill-adults [31]. But the evidence lacks how frequent these strategies should be implemented in CICU settings [25].

Delirium progression in the ICU has been associated with sedation and intubation of patients. Earlier studies have proven that frequent interruptions to sedative medications allowing them to wake up and spontaneous breathing strategies have shown decreased mechanical ventilation duration by two days (p=0.004) and decrease in weaning time also by two days (p=0.003) [32].

**Figure 2:**
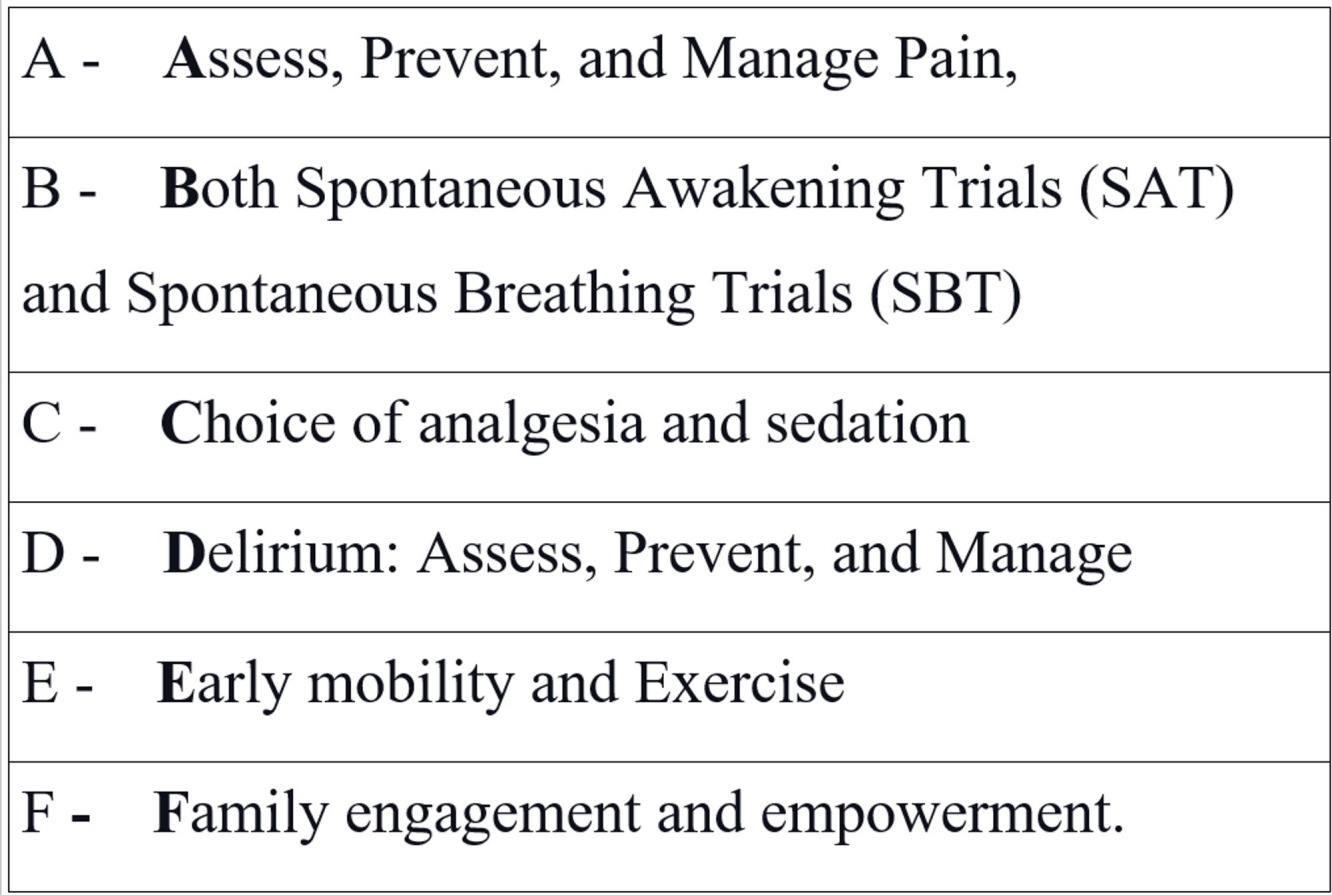
ABCDEF Bundle SAT: Spontaneous Awakening Trials, SBT: Spontaneous Breathing Trials

A multicenter cohort study of 15,226 adults in the ICU by Pun et al. demonstrated that complete ABCDE bundle (Figure 2) implementation is associated with a lower likelihood of hospital death within seven days (adjusted hazard ratio, 0.32; CI, 0.17-0.62), next-day mechanical ventilation (adjusted odds ratio (AOR), 0.28; CI, 0.22-0.36), coma (AOR, 0.35; CI, 0.22-0.56), delirium (AOR, 0.60; CI, 0.49-0.72), physical restraint use (AOR, 0.37; CI, 0.30-0.46), ICU readmission (AOR, 0.54; CI, 0.37-0.79), and discharge to a facility other than home (AOR, 0.64; CI, 0.51-0.80) [33].

Focus on early mobilization in the ICU patients comes with the concern of their safety and efficacy, involvement, and skepticism that they are too sick for participation, but recent studies challenged by implementing early rehabilitation in ICU patients at more initial stages of their stay. Schweickert et al. demonstrated that patients who were randomized to receive early rehabilitation during their ICU stay experienced shorter delirium periods, i.e., two days vs. four days with no rehabilitation (p=0.02) [34]. Occupational therapy (OT) may also be beneficial in cardiac-ICU patients. A recent study showed a lower delirium incidence in patients with OT exposure than no OT (3% versus 20%, p<0.001) [25]. A study performed by combining physical therapy (PT), OT and low-dose benzodiazepines in medical ICU patients marked a reduced incidence of delirium (21% vs. 53%, p=0.003) and a reduction in hospital stay by 3.1 days (95% CI; 0.3-5.9 days) [25]. Even with the need for further research on the topic, it is prudent to consider that PT and OT are beneficial to ICU patients. It may be challenging to implement these in cardiac ICU patients because of the patients' complexity and added mechanical assistance devices. However, every attempt should be made to implement PT and OT in cardiac ICU patients at the early stages of their stay for beneficial results.

For ICU patients, sleep deprivation is a critical hurdle in which non-pharmacological management strategies become essential. Several techniques, such as reducing the alarm volumes, closing the doors, eye masks, earplugs, and timed turning off lights, have been proved to enhance the patient's sleep in the ICU [1,25]. A systematic review of 10 studies assessed delirium relation after sleep interventions and noticed six studies demonstrated a statistically significant reduction in ICU delirium incidence. Four studies evaluated the duration of delirium, of which three reported a shorter duration of delirium with sleep intervention-two studies associated sleep intervention with a reduced ICU length of stay [35].

Pharmacological management

Benzodiazepines

Benzodiazepines are associated with an increased risk of delirium (OR = 3.0, 1.3-6.8) [36]. So, a comprehensive search for alternative sedatives mounted, and the use of propofol was evaluated because of its short-acting properties. It has shown better results compared to midazolam by reducing the length of mechanical ventilation [25]. Two studies compared propofol with benzodiazepines and showed a shorter time to light sedation with propofol. Seven randomized controlled trials (RCTs) considered a shortened time to extubation to be clinically significant with propofol [31]. Based on these findings, the Society of Critical Care Medicine proposed practical guidelines, with low quality of evidence that propofol is preferred to benzodiazepines for sedation in mechanically ventilated adults after cardiac surgery [31]. However, more RCTs are required comparing benzodiazepines and propofol to evaluate other critical outcomes in the cardiac surgical population. 

Antipsychotics

Antipsychotic use as a preventive or therapeutic agent in delirium is acknowledged because of the limitations of using benzodiazepines in delirium patients. But the use of these medications has been uncertain and was associated with limited to no benefit. A large meta-analysis study comparing antipsychotics to placebo or no interventions reported no significant antipsychotics effect on delirium incidence (OR = 0.56, 0.23-1.34) [37]. It is also vital to consider the most striking adverse effect of these medication use in cardiovascular patients, which is QT-prolongation and increased mortality risk in demented elderly patients thus resulted in a black-boxed warning by the Food and Drug Administration [25,38] 

Dexmedetomidine

Dexmedetomidine, an alpha-2 agonist, has sedative properties with an insignificant effect on the respiratory drive and also reduces delirium by more direct neuroprotective mechanisms [25,31]. These mechanisms have led to many clinical investigations involving dexmedetomidine in the ICU. Table 3 summarizes the various studies that have shown the significant role dexmedetomidine plays in reducing the risk of delirium developing in cardiac ICU patients. 

**Table 2:** Summarizes a few studies evaluating dexmedetomidine in reducing the risk of delirium in the surgical and ICU population. DEX: dexmedetomidine, MDX: Midazolam, POD: Postoperative delirium

Study	Study population	Study Comparison	Delirium incidence/outcomes	Notes
SEDCOM trial, 2009, [39]	375 surgical ICU patients	DEX vs. MDX	DEX vs. MDX (54% vs 76.6%; p<0.001)	Less delirium POD with DEX
Deiner et al. 2016, [40]	404 surgical patients	Intraoperative DEX vs. NS	DEX vs. NS (12.2% vs 11.4%; p = 0.94)	No difference in POD incidence.
Su X et al., 2016, [41]	700 non-cardiac surgery patients	Postoperative DEX vs. Placebo	DEX vs. Placebo Odds ratio (9% vs 23%; p<0.0001)	Decrease in POD risk with DEX
Shehabi et al.,2009, [42]	306	Postoperative DEX vs. Morphine	DEX vs. Morphine (8.6% vs 15%; p<0.088)	No decrease in incidence but decrease in median duration
Liu X et al., 2017, [43]	Meta-Analysis of 8 studies	DEX vs. Propofol sedation after cardiac surgery	DEX vs. Propofol (Risk ratio, 0.40; P=.0002),	DEX decreases POD and shorter intubation

Statins

The concept of statin use in delirium patients is controversial. The hypothesis behind using statins in delirium is its anti-inflammatory phenotype properties in animal studies that may help neuronal healing rather than neuronal apoptosis by microglial activation [32]. However, a recent meta-analysis observed no favorable effect concerning statins for delirium prevention (RR, 1.03 (95% CI, 0.68-1.56); p=0.89) [44].

Limitations

This study, however, is subject to a few limitations. Animal studies and the literature published primarily in languages other than English were not considered for this review. Also, many studies in the review are not entirely studied in CICU settings, and reflecting the ICU data and generalizing it with CICU is not always a unique approach.

## Conclusions

Delirium is a multifactorial syndrome, and the erroneous view of delirium as an inevitable consequence of patients' illness in the healthcare team should be discouraged. Every effort should be made to minimize the modifiable risk factors of delirium, especially in high-risk patients. Clinicians should be aware of the disease's broad presentation to effectively identify and manage delirium in clinical settings. Implementing effective delirium screening methods is possible with a multidimensional team approach involving all the healthcare members involved in the patient's care, such as nursing-assessments, medical students, and resident physicians' rounds. Screening tools require minimal time and are cost-effective, so they should be performed in all CICU patients at the early stages of their admission.

Due to the limitations in generalizing the standard ICU data to the CICU, it is evident that substantial research is needed to agree or disagree with the present data. More studies are required to provide the effectiveness of early rehabilitation, occupational therapy in CICU patients. More significant clinical trials are necessary to evaluate dexmedetomidine's safety and efficacy, antipsychotics, and statin use in CICU patients with complex cardiovascular diseases. 
